# Why Have Tobacco Control Policies Stalled? Using Genetic Moderation to Examine Policy Impacts

**DOI:** 10.1371/journal.pone.0050576

**Published:** 2012-12-05

**Authors:** Jason M. Fletcher

**Affiliations:** Department of Health Policy and Management, Yale School of Public Health, New Haven, Connecticut, United States of America; Sanjay Gandhi Medical Institute, India

## Abstract

**Background:**

Research has shown that tobacco control policies have helped produce the dramatic decline in use over the decades following the 1964 surgeon general’s report. However, prevalence rates have stagnated during the past two decades in the US, even with large tobacco taxes and expansions of clean air laws. The observed differences in tobacco control policy effectiveness and why policies do not help all smokers are largely unexplained.

**Objective:**

The aim of this study was to determine the importance of genetics in explaining response to tobacco taxation policy by testing the potential of gene-policy interaction in determining adult tobacco use.

**Methods:**

A moderated regression analysis framework was used to test interactive effects between genotype and tobacco policy in predicting tobacco use. Cross sectional data of US adults from the National Health and Nutrition Examination Survey (NHANES) linked with genotype and geocodes were used to identify tobacco use phenotypes, state-level taxation rates, and variation in the nicotinic acetylcholine receptor (*CHRNA6*) genotype. Tobacco use phenotypes included current use, number of cigarettes smoked per day, and blood serum cotinine measurements.

**Results:**

Variation in the nicotinic acetylcholine receptor was found to moderate the influence of tobacco taxation on multiple measures of tobacco use. Individuals with the protective G/G polymorphism (51% of the sample) responded to taxation while others had no response. The estimated differences in response by genotype were C/C genotype: b = −0.016 se  = 0.018; G/C genotype: b = 0.014 se  = 0.017; G/G genotype: b = −0.071 se 0.029.

**Conclusions:**

This study provides novel evidence of “gene-policy” interaction and suggests a genetic mechanism for the large differences in response to tobacco policies. The inability for these policies to reduce use for individuals with specific genotypes suggests alternative methods may be needed to further reduce use.

## Introduction

Tobacco use is among the most important causes of morbidity and the leading preventable cause of death in the US, with over 400,000 deaths per year [1]. This number accounts for more deaths than AIDS, alcohol use, cocaine use, heroin use, homicides, suicides, motor vehicle crashes, and fires combined [2], [3]. Although studies suggest that as much as 70% of the variance in nicotine dependence and other tobacco use phenotypes could be due to genetic factors [4], the principal policies to reduce tobacco use have been broad-based and non-targeted. Following economic theory, governments have sought to increase the price of tobacco in order to reduce consumption [5]. Indeed, one of the most successful policies to reduce tobacco use has been tobacco taxation [6], helping to reduce use by over 50% since the mid 1960 s, which has been suggested as one of the most successful public health interventions in the 20^th^ century [3], [7]. Following recommendations from the IOM and other groups [3], recent large increases in tobacco taxes have been made. For example, in April 2009, the largest federal excise tax in history went into effect, bringing the average combined federal and state rates to over $2 per pack [8].

However, these large changes in policy have not produced commensurate reductions in tobacco use, which has been largely unchanged for the past 20 years, varying between 20% and 25%. Indeed, there has been important heterogeneity of the impacts of the policy between broadly defined socio-demographic groups, such as by age, gender, race, and socioeconomic status [9], [10]. There is also emerging evidence that tax responses may be related to self control and other characteristics [11]. The sources of these heterogeneous responses are not understood by social scientists or policymakers interested in both further reductions in use as well as enriching theoretical understandings of the complex determinants of use and cessation. Specifically, the large differentials in responses have not been fully examined or elucidated in order to predict individual differences and discover why taxation does not seem to work for everyone.

Because of the prominence of genetic factors in determining tobacco use phenotypes [4], a remaining question is whether these genetic factors are an important source of the success (and failure) of these broad-based tobacco control policies. In order to examine genetic heterogeneity in the response to policy, this paper targets a specific polymorphism in the alpha-6 subunit found in nicotinic acetylcholine receptors found primarily in the brain and expressed on dopamine-releasing neurons in the midbrain [12]. This variant was selected because it directly mediates the rewarding aspects of nicotine consumption, and does not play a primary role in mediating the effects of other drugs of abuse. The hypothesis of interest in this study is whether this polymorphism interacts with a specific environmental exposure–state-level tobacco tax rates–in determining tobacco use. This interaction would produce novel evidence of potential gene-policy interaction (GxP) and increase understanding of why tobacco taxation produces heterogeneous responses.

## Methods

This gene-policy interaction hypothesis is tested using four years of cross sectional data from the National Health and Nutrition Health Examination III (NHANES) Phase 2 study (1991–1994). This survey is representative of the civilian non-institutionalized US population ages 2 months and older. Military personnel, nursing home residents, prisoners and others are excluded. The data consists of repeated-cross sectional surveys of approximately 10,000 adults. Data collection included survey responses as well as biological specimens, and recent and ongoing genotyping of these archived data has begun for approximately 7,200 respondents. In unreported results, I find no differences in the likelihood of contributing DNA data by race, gender, or income levels. For the analyses reported here, individuals are divided based on their *CHRNA6* genotype (C/C, C/G, G/G in the rs2304297 SNP). The proportions of individuals were 11%, 38%, and 51%, respectively. Although now 20 years old, the NHANES is the only suitable available database to examine the question of interest on a national adult population, and the rates of adult smoking in the early 1990 s are only slightly higher than those in current years.

*CHRNA6* provides a logical candidate gene for modulating variation in tobacco use. It has a well replicated role in tobacco use and nicotine dependence [13]–[16], which is supported by studies of the biological mechanisms of *CHRNA6* using mouse models [17]. Further, information on a commonly studied SNP within this gene (rs2304297) is available in the novel and opportunistic series of studies on US adults (NHANES). It was also the only available known tobacco-specific SNP included in the NHANES III data. As shown in previous research [14]–[16] and replicated here, the G/G genotype is related to lower likelihood of tobacco use in the nationally representative sample of US adults surveyed for this study (**Table S1**). This association likely operates through an addiction pathway, as several studies have linked this genotype with nicotine dependence DSM-IV criteria [16].

The phenotypes of interest relate to current tobacco use and are assessed through self report as well as laboratory-based serum cotinine levels (ng/ml). Twenty five percent of the sample reported being a current smoker. Of those who reported being a current smoker, over ninety percent of their measured cotinine values supported their self reports, and results will be presented for both measures. The environmental exposure of interest is the state-level per-pack tobacco tax rate, which on average for these years was nearly $0.25 and varied from $0.02 to $0.56 across states. The rates are matched to the data at the state and year-levels and are not adjusted for inflation for the four years of data. See **Table 1** for full descriptive statistics for the analysis sample. See the **Materials & Methods S1** for additional information on the Methods of the study.

**Table 1:** Summary Statistics.

Variable	Mean/Proportion	Std Dev	Min	Max
Tobacco Use (Binary)	0.25		0	1
Number of Cigarettes Daily	3.73	8.76	0	140
Number of Cigarettes Daily (Among Smokers)	15.2	11.78	1	140
Cotinine (ng/ml)	68.41	139.29	0.35	1890
Age (years)	42.83	17.09	17	90
Female	0.52		0	1
White Race	0.75		0	1
Black race	0.10		0	1
Hispanic ethnicity	0.10		0	1
Other Race	0.05		0	1
Education (years)	12.52	3.06	0	17
Income ($1000 s)	35.32	18.39	0	60
Married	0.61		0	1
Missing Information on Demographic Characteristics	0.06		0	1
State Level Tobacco Use Rate	0.25	0.04	0.17	0.34
Cigarette Tax Level (cents per pack)	24.53	11.34	2.0	56.0
SNP:				
rs2304297 = = “CC”	0.11		0	1
rs2304297 = = “CG”	0.38		0	1
rs2304297 = = “GG”	0.51		0	1

NHANES 1991–1994 Genetic Sample (N = 6,178).

Notes: Author’s calculations from NHANES Data. Sample weights used.

### Statistical Analysis

The study used a moderated regression framework [18] to test the association between current tobacco use and (i) *CHRNA6* genotype, (ii) the logarithm of state-level tobacco tax rates and (iii) their interaction. The baseline empirical model had no other covariates:
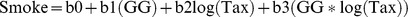
where: b0 is the intercept; b1 is the regression coefficient associated with the effect of variation in the *CHRNA6* gene (in the rs2304297 SNP), which is coded as GG = 1 and CC/GC  = 0; b2 is the coefficient associated with effect of the logarithm of the state-level tobacco tax rate; b3 is the coefficient associated with the interaction effect, which is the product of the two variables (genotype and tax). The logarithm of state-level taxes is used to conform to prior research and to facilitate comparison of the results [19], [20]. Current tobacco use is used as the primary outcome due to the cross sectional nature of the data, as the timing of initiation and quitting behavior is unknown. An extended model statistically adjusted for covariates, including age, age-squared, gender (Female = 1), Race/ethnicity indicator variables (Black = 1, Hispanic = 1, White = 0), years of schooling (continuous), current family income ($1000 s), marital status (Married =  = 1), and an indicator for whether any sociodemographic variables were missing. As a second extension, to reduce population stratification, a specification was estimated that only included individuals who self-reported “white” race (**Table S3**).

All models used ordinary least squares regression due to the known issues with properly estimating interaction effects in non-linear models, such as logistic regression [21] and the simplicity of estimating linear models when the outcome is not near the 0/1 boundary as it is for the smoking rate in this data (prevalence is 25%). The full results of these regressions are provided in **Table 2**. Due to the hierarchical nature of the data (individuals nested within states), robust standard errors are clustered for arbitrary non-independence at the state level in all analyses. Sample weights provided by NHANES are used throughout in order to produce nationally representative estimates.

**Table 2:** Gene-Environment Interactions in Predicting Tobacco Use.

Outcome	Tobacco Use	Tobacco Use
Specification	Interaction Only	Additional Covariates
Log (Tax)	0.002	0.016
	(0.013)	(0.014)
rs2304297 = = G/G	−0.037**	−0.032*
	(0.017)	(0.018)
SNP X Log (Tax)Interaction	−0.073***	−0.072***
	(0.024)	(0.024)
Age		0.016***
		(0.003)
Age-squared		−0.000***
		(0.000)
Female		−0.064***
		(0.018)
Black		−0.062**
		(0.022)
Hispanic		−0.184***
		(0.046)
Other Race		−0.101*
		(0.053)
Education		−0.024***
		(0.004)
Income ($1000 s)		−0.003***
		(0.001)
Married		−0.024*
		(0.013)
Missing Information		0.011
		(0.043)
Constant	0.270***	0.458***
	(0.018)	(0.111)
Observations	6178	6178
R-squared	0.007	0.094

Robust standard errors in parentheses clustered at the state level.

*** p<0.01,

** p<0.05,

* p<0.1.

Sample weights used.

Notes: Results for regression analyses testing GXE interaction effects on tobacco use reports. This table presents the final results where the main and interaction effects are entered simultaneously. All results use linear probability models (LPM), which is an ordinary least squares (OLS) regression predicting a binary variable outcome (Smoke  = 0/1). The main result is found in Column 2, where the statistical interaction between individual G/G genotype and state level tax rates is negative and statistically significant (P = <0.01). The regression coefficient for Log(Tax) suggests that individuals with the C/C or C/G genotypes are not responsive to higher rates of tobacco taxation. The regression coefficient for the G/G genotype suggests that individuals with this genotype are less likely to report current tobacco use than individuals with the C/G or C/C genotypes. The regression coefficient for the Interaction (G/G X Log (Tax)) suggests that a 10% tax increase reduces the likelihood of reported tobacco use for those with the G/G genotype by 0.73 percentage points more than those with C/G or C/C genotypes. Column 3 includes addition demographic in the analysis to test the robustness of the coefficient on Interaction. See Statistical Analysis section for further details.

### Gene-Environment Interaction Results

**Figure 1:**
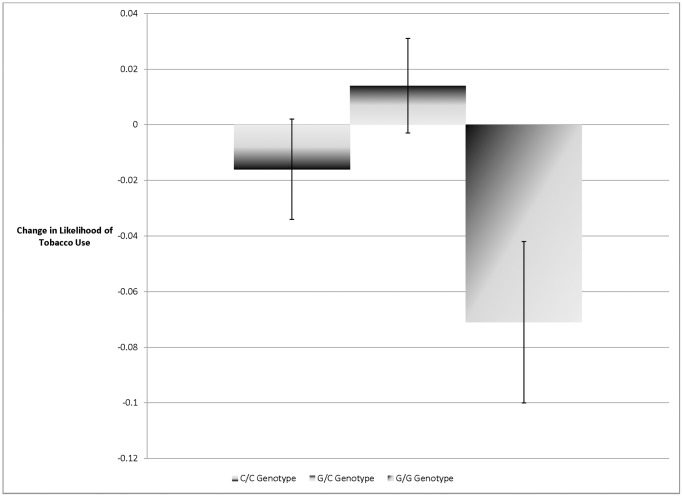
Predicted Reductions in the Likelihood of Current Tobacco Use Based on a 100% Increase in State Tobacco Tax Rates: Results Stratified by *CHRNA6* Genotype. Notes: Author’s calculation from NHANES Data. Results from three separate regression analyses estimating the association between state level tobacco tax rates and tobacco use based on *CHRNA6* genotype (C/C, G/C, G/G). Among the 1,278 individuals with C/C genotype, the estimated tax effect was not significant (b = −0.016, SE = 0.018, z = .89, P = 0.37). Among the 2,328 individuals with the G/C genotype, the estimated tax effect was not significant (b = 0.014, SE = 0.017, z = .82, P = .41). However, among the 2,572 individuals with the G/G genotype, the estimated tax effect was statistically significant (b = −0.071, SE = 0.029, z = 2.44, P = 0.0148). These effects were statistically different based on genotype (see full results in **Table 2**). This interaction showed that only adults with the G/G genotype respond to tobacco taxes in the manner predicted by economic theory. Robust standard error bars clustered at the state level. Sample weights were used in order to produce nationally representative estimates.

This study used a moderated regression framework to test the association between current tobacco use and (i) *CHRNA6* genotype, (ii) the logarithm of state-level tobacco tax rates and (iii) their interaction. These results are reported in **Table 2**. Like previous literature, the G/G genotype is protective, as are high tobacco taxes; the interaction between *CHRNA6* and tax rates showed that the effect of the tax is only present for individuals with the protective (G/G) genotype (p<.01) and absent in other individuals. **Figure 1** provides the estimated differences in tobacco tax response stratified by genotype (C/C genotype: b = −0.016 se  = 0.018; G/C genotype: b = 0.014 se  = 0.017; G/G genotype: b = −0.071 se 0.029). **Table 2** **, Column 3 (“Additional Covariates”)** shows that these results are robust to the addition of demographic covariates. While there are other risk factors related to tobacco use not available in the NHANES data, these factors would need to be also related to this genotype in order to confound the findings here. Results showing that the genotype is unrelated to gender, age, education, income, and marital status, among other variables, are available from the author.

The study then used the same moderated regression to test associations between quantity of cigarettes smoked and also laboratory-tested cotinine levels. In both cases, the findings suggest an interaction between the G/G genotype and the logarithm of state tobacco tax rates (p<0.01) (**Table S2**). In order to examine issues of population stratification, the results were estimated with the sub-sample of respondents who reported “white” race. The results were nearly identical and shown in **Table S3**.

An important limitation in detecting causal G X E effects in past work is potential gene-environment correlation, where genotype is related to the risk of exposure, potentially through unmeasured gene-gene interaction [22], [23]. For example, a genotype that places a child at risk for depression (and which was inherited from parents) may also place the child a risk of experiencing a parental divorce. Interacting this “stressful life event” with genotype might then be confounded through gene-environment correlation. However, there is little reason to believe that genotype would be related to state level taxation levels [24]. Indeed, there were no statistically significant differences in the tax rates faced by individuals based on genotype–See **Table S4.** Finding no discernible differences based on genotype reduces the likelihood of residual confounding at the state-level, as potential confounders related to state taxation would produce correlations between state taxation levels and genotype. This suggests an added likelihood of the estimation of causal effects and a relative strength over existing studies.

## Discussion

This study showed that the impact of tobacco taxation on tobacco use is moderated by individual genotype. Indeed, only individuals with the protective G/G genotype were found to respond to state level tobacco taxation rates, suggesting an important clue in understanding why the rates of tobacco use among US adults have remained stubbornly persistent during a time period of large changes in tobacco control policies during the past two decades.

Unlike previous reports of G X E, where individuals might have a heritable tendency to have an elevated likelihood of being exposed to a particular “environment”, the current application has minimal issues with so-called gene-environment correlation because the *CHRNA6* genotype is unrelated to state-tax policy. Relative to prior studies, this allows greater certainty in establishing causal interactive effects between genotype and exposure. Additionally, the use of a policy instrument to examine modulation of genetic effects is novel and more easily subject to intervention than alternative measures of environmental exposure (e.g. divorce, child maltreatment), even if the policy cannot be targeted to certain genotypes.

Like previous reports of G X E, until this study’s findings are replicated in alternate samples, policy recommendations are premature. In particular, it would be of interest to test whether higher tax levels recently introduced in the US and other developed countries may have different effectiveness on the unresponsive genotypes in the current study. These findings also suggest a need for further understanding of the functional properties of the *CHRNA6* and associated genes, which could potentially be used to create pharmacological treatments for current smokers who may be unresponsive to major health policy interventions, such as tobacco taxation. Indeed, studies suggest that rs2304297 may not be the functional allele, but may instead be in linkage disequilibrium with the actual disease allele, particularly within the LD block that extends beyond exon 4 in *CHRNA6* [16]. Other nicotinic acetylcholine receptors have been associated with nicotine addiction [12], [17], [25]–[27], so this variant may also operate in collaboration with similar surrounding genes. An alternative hypothesis would be that this polymorphism may affect expression through trafficking of mRNA, miRNA binding or rate of desegregation [28], [29].

If replicated, the findings in this study are suggestive of a key and under-examined genetic role in determining response to important health policies. The results are stark in that a single SNP is used to completely segment the population into the approximately 50% of adults who are likely to respond to tobacco taxation and the 50% who are unresponsive. This is an important first step in future health policy efforts to further reduce adult smoking rates. Additionally, this study has begun a new examination of potential gene X policy (G X P) interactions that may have broad scope in learning why some policies are effective and other are not and also deepen our understanding of the genetic response to broad-based policy interventions.

## Supporting Information

Table S1 **Gene-Environment Interactions in Predicting Tobacco Use.** Robust standard errors in parentheses clustered at the state level. *** p<0.01, ** p<0.05, * p<0.1 Notes: This table reports the statistical associations between the tobacco use (the outcome) and state level tobacco tax rates (Column 1), individual’s genotype (Column 2), and both variables together (Column 3). Column 1 reports unadjusted differences in tobacco tax use as predicted by the logged values of the tobacco tax and shows that a 100% increase in the tax rate is associated with a 3.1 percentage point reduction in the likelihood of reporting tobacco use. Column 2 reports the unadjusted differences in tobacco use as predicted by genotype and shows that individuals with the G/G genotype are 3.7 percentage points less likely to report current tobacco use than individuals with C/C or C/G genotype. Column 3 reports the likelihood of tobacco use as predicted by both genotype and tobacco tax rate and shows similar results as Columns 1 and 2.
(DOCX)

Table S2 **Gene-Environment Interactions in Predicting Tobacco Use.** Alternative Measures of Tobacco Use. Robust standard errors in parentheses clustered at the state level. *** p<0.01, ** p<0.05, * p<0.1. Sample weights used. Notes: Results for regression analyses testing GXE interaction effects on two measures of tobacco use (1) the reported typical number of cigarettes smoked per day and (2) the serum cotinine level from laboratory assessment. This table presents the final results where the main and interaction effects are entered simultaneously. All results use linear ordinary least squares (OLS) regression. See Statistical Analysis section and Notes for Table S1 for further details.
(DOCX)

Table S3 **Gene-Environment Interactions in Predicting Tobacco Use.** White Race Only. Robust standard errors in parentheses clustered at the state level. *** p<0.01, ** p<0.05, * p<0.1. Sample weights used. Notes: Results for regression analyses testing GXE interaction effects on tobacco use reports. This table presents the final results where the main and interaction effects are entered simultaneously and the sample only includes individuals who reported “white” race in the survey. All results use linear probability models (LPM), which is an ordinary least squares (OLS) regression predicting a binary variable outcome (Smoke  = 0/1). See Statistical Analysis section and Notes from Table S1 for further details.
(DOCX)

Table S4 **Adjusted Correlations between Genotype and State Level Tax Levels.** Robust standard errors in parentheses clustered at the state level in columns 2 and 3. *** p<0.01, ** p<0.05, * p<0.1. Sample weights used. Notes: This table reports the statistical associations between the state level tobacco tax rate (the outcome) and individual’s genotype. Column 1 reports unadjusted differences in the tax rate based on genotype while Column 2 reports the statistically adjusted differences. For column 1, the interpretation is that individual with the C/G genotype are exposed to tobacco rates that are not statistically different (b = 1.985, SE = 1.449) than individuals with C/C genotype, which is the omitted comparison group. The R-squared calculation suggests no more than 0.4% of the variance in tobacco tax rates can be accounted for by differences in genotype.
(DOCX)

Materials & Methods S1 (DOCX)
