# Supplementary material for: Why Have Tobacco Control Policies Stalled? Using Genetic Moderation to Examine Policy Impacts
Source: PLoS One. 2012 Dec 5;7(12):e50576. doi: 10.1371/journal.pone.0050576 (PMC3515624; doi:10.1371/journal.pone.0050576)
Supplement: Table S1 — Gene-Environment Interactions in Predicting Tobacco Use. Robust standard errors in parentheses clustered at the state level. *** p<0.01, ** p<0.05, * p<0.1 Notes: This table reports the statistical associations between the tobacco use (the outcome) and state level tobacco tax rates (Column 1), individual’s genotype (Column 2), and both variables together (Column 3). Column 1 reports unadjusted differences in tobacco tax use as predicted by the logged values of the tobacco tax and shows that a 100% increase in the tax rate is associated with a 3.1 percentage point reduction in the likelihood of reporting tobacco use. Column 2 reports the unadjusted differences in tobacco use as predicted by genotype and shows that individuals with the G/G genotype are 3.7 percentage points less likely to report current tobacco use than individuals with C/C or C/G genotype. Column 3 reports the likelihood of tobacco use as predicted by both genotype and tobacco tax rate and shows similar results as Columns 1 and 2. (DOCX) [file pone.0050576.s001.docx]

Table S1

| Outcome | Tobacco Use | Tobacco Use | Tobacco Use |
| --- | --- | --- | --- |
| Specification | Tax Only | Gene Only | Both |
| Log (Tax) | -0.031* |  | -0.030* |
|  | (0.017) |  | (0.016) |
| rs2304297==G/G |  | -0.037* | -0.035* |
|  |  | (0.019) | (0.020) |
| Constant | 0.251*** | 0.270*** | 0.268*** |
|  | (0.015) | (0.017) | (0.018) |
|  |  |  |  |
| Observations | 6178 | 6178 | 6178 |
| R-squared | 0.002 | 0.002 | 0.004 |
