# Supplementary material for: Why Have Tobacco Control Policies Stalled? Using Genetic Moderation to Examine Policy Impacts
Source: PLoS One. 2012 Dec 5;7(12):e50576. doi: 10.1371/journal.pone.0050576 (PMC3515624; doi:10.1371/journal.pone.0050576)
Supplement: Table S2 — Gene-Environment Interactions in Predicting Tobacco Use. Alternative Measures of Tobacco Use. Robust standard errors in parentheses clustered at the state level. *** p<0.01, ** p<0.05, * p<0.1. Sample weights used. Notes: Results for regression analyses testing GXE interaction effects on two measures of tobacco use (1) the reported typical number of cigarettes smoked per day and (2) the serum cotinine level from laboratory assessment. This table presents the final results where the main and interaction effects are entered simultaneously. All results use linear ordinary least squares (OLS) regression. See Statistical Analysis section and Notes for Table S1 for further details. (DOCX) [file pone.0050576.s002.docx]

Table S2

| Outcome | Number of Cigarettes | Cotinine |
| --- | --- | --- |
|  |  |  |
| Log (Tax) | 0.139 | -3.046 |
|  | (0.344) | (3.129) |
| rs2304297==G/G | -0.25 | 0.063 |
|  | (0.370) | (6.789) |
| Interaction | -0.782*** | -12.188*** |
|  | (0.265) | (4.146) |
| Age | 0.511*** | 5.792*** |
|  | (0.065) | (0.939) |
| Age-squared | -0.006*** | -0.068*** |
|  | (0.001) | (0.010) |
| Female | -2.121*** | -30.823*** |
|  | (0.429) | (5.472) |
| Black | -3.811*** | -2.145 |
|  | (0.649) | (5.729) |
| Hispanic | -5.894*** | -70.994*** |
|  | (1.106) | (15.340) |
| Other Race | -3.189*** | -27.386 |
|  | (0.876) | (17.451) |
| Education | -0.557*** | -8.777*** |
|  | (0.107) | (1.749) |
| Income ($1000s) | -0.072*** | -0.596*** |
|  | (0.018) | (0.193) |
| Married | -0.219 | -5.721 |
|  | (0.330) | (4.517) |
| Missing Information | 0.073 | -8.33 |
|  | (0.842) | (10.750) |
| Constant | 7.880** | 121.797*** |
|  | (2.753) | (39.737) |
|  |  |  |
| Observations | 6,130 | 6,089 |
| R-squared | 0.096 | 0.085 |
