# Supplementary material for: Why Have Tobacco Control Policies Stalled? Using Genetic Moderation to Examine Policy Impacts
Source: PLoS One. 2012 Dec 5;7(12):e50576. doi: 10.1371/journal.pone.0050576 (PMC3515624; doi:10.1371/journal.pone.0050576)
Supplement: Table S3 — Gene-Environment Interactions in Predicting Tobacco Use. White Race Only. Robust standard errors in parentheses clustered at the state level. *** p<0.01, ** p<0.05, * p<0.1. Sample weights used. Notes: Results for regression analyses testing GXE interaction effects on tobacco use reports. This table presents the final results where the main and interaction effects are entered simultaneously and the sample only includes individuals who reported “white” race in the survey. All results use linear probability models (LPM), which is an ordinary least squares (OLS) regression predicting a binary variable outcome (Smoke = 0/1). See Statistical Analysis section and Notes from Table S1 for further details. (DOCX) [file pone.0050576.s003.docx]

Table S3

| Outcome | Tobacco Use |
| --- | --- |
| Sample | White |
| Log (Tax) | 0.011 |
|  | (0.020) |
| Genotype = GG | -0.028 |
|  | (0.022) |
| Interaction | -0.082*** |
|  | (0.025) |
| Observations | 2,372 |
| R-squared | 0.008 |
