# Supplementary material for: Why Have Tobacco Control Policies Stalled? Using Genetic Moderation to Examine Policy Impacts
Source: PLoS One. 2012 Dec 5;7(12):e50576. doi: 10.1371/journal.pone.0050576 (PMC3515624; doi:10.1371/journal.pone.0050576)
Supplement: Table S4 — Adjusted Correlations between Genotype and State Level Tax Levels. Robust standard errors in parentheses clustered at the state level in columns 2 and 3. *** p<0.01, ** p<0.05, * p<0.1. Sample weights used. Notes: This table reports the statistical associations between the state level tobacco tax rate (the outcome) and individual’s genotype. Column 1 reports unadjusted differences in the tax rate based on genotype while Column 2 reports the statistically adjusted differences. For column 1, the interpretation is that individual with the C/G genotype are exposed to tobacco rates that are not statistically different (b = 1.985, SE = 1.449) than individuals with C/C genotype, which is the omitted comparison group. The R-squared calculation suggests no more than 0.4% of the variance in tobacco tax rates can be accounted for by differences in genotype. (DOCX) [file pone.0050576.s004.docx]

Table S4

| Outcome | State Tax Level | State Tax Level |
| --- | --- | --- |
| Genotype = C/C | Omitted |  |
|  |  |  |
| Genotype = C/G | 1.985 | 0.58 |
|  | (1.449) | (0.574) |
| Genotype = G/G | 2.143 | 0.478 |
|  | (1.796) | (0.708) |
| Age |  | 0.017 |
|  |  | (0.027) |
| Black |  | -1.771 |
|  |  | (2.486) |
| Hispanic |  | 8.807*** |
|  |  | (2.224) |
| Other Race |  | 2.402 |
|  |  | (1.753) |
| Education |  | 0.243 |
|  |  | (0.159) |
| Income |  | 0.027 |
|  |  | (0.029) |
| Year = 1992 |  | 13.757*** |
|  |  | (4.051) |
| Year = 1993 |  | 15.542*** |
|  |  | (2.783) |
| Year = 1994 |  | 13.482*** |
|  |  | (3.371) |
| Constant | 26.096*** | 7.815** |
|  | (3.697) | (3.093) |
|  |  |  |
| Observations | 7,008 | 6,178 |
| R-squared | 0.004 | 0.113 |
