# Supplementary material for: Why Have Tobacco Control Policies Stalled? Using Genetic Moderation to Examine Policy Impacts
Source: PLoS One. 2012 Dec 5;7(12):e50576. doi: 10.1371/journal.pone.0050576 (PMC3515624; doi:10.1371/journal.pone.0050576)
Supplement: Materials & Methods S1 — (DOCX) [file pone.0050576.s005.docx]

Supplementary Material

Why Have Tobacco Control Policies Stalled? Using Genetic Moderation to Examine Policy Impacts

**Materials and Methods**

Research sample: Participants were respondents to the National Health and Nutrition Surveys (NHANES) III, 1991-1994 (Phase II). The NHANES is a program of studies designed to assess the health and nutritional status of adults and children in the United States and collected by the Centers for Disease Control and Prevention.^[[1]](#footnote-1)^ NHANES III began in the Fall of 1988 and ended in the Fall of 1994. Survey data were collected and can be analyzed from two phases: Phase-1 was conducted from October, 1988 to October, 1991, and Phase-2 was conducted from October, 1991 to October, 1994. Both phases are nationally representative samples.

To add to the extensive amount of information collected for the purpose of describing the health of the population, DNA specimens were collected during the NHANES III surveys. The genetic data available through the NCHS is from 7,159 specimens collected during Phase-2 of NHANES III. This collection is an ongoing process and additional genetic variation information become available every six months^[[2]](#footnote-2)^. At the time of this study, *CHRNA6* (rs2304297) was the only gene related to only tobacco use in the dataset. This study uses 6,178 adults who were genotyped for the *CHRNA6* gene and had non-missing information for state of residence and smoking status.

*CHRNA6*: The *CHRNA6* (Cholinergic receptor, nicotinic, alpha 6) gene encodes an alpha subunit of neuronal nicotinic acetylcholine receptors. This gene is among a class of genes, which are the primary targets for nicotine in the brain, involved in nicotine-related behaviors and is in the family of nicotinic acetycholine receptors (nAChRs), where each subunit is encoded by a single gene (S1).^^[[3]](#footnote-3)^^ ^^[[4]](#footnote-4)^^ In addition to research using animal models, several studies have linked *CHRNA6* with tobacco use phenotypes (S2-S5).

DNA extraction and genotyping: No genotyping was completed by the author of this study. The CDC has produced the following description: “The laboratory distributes DNA as aliquots of crude cell lysates. DNA concentrations vary and are estimated to range from 7.5–65 ng/µL with an average of approximately four micrograms in 100 ul. Each 96 well plate is bar-coded and labeled with a readable identifier. Quality control samples (approximately 480 samples) are sent at no charge, either inserted with the NHANES samples or in separate plates, as blind replicates and/or blanks.”^[[5]](#footnote-5)^

Population stratification cannot be completely ruled out as a confounding factor in this study. To limits its likely effect, this study statistically controls for self-reported race and includes results only for individuals who self-report as “white” (Table S3).

State-level tobacco taxes: the environmental exposure in this study was the tax levied by the state (in cents) for each pack of cigarettes purchased. These rates were gathered from a secondary source (S6). There were no statistically significant differences in tax rates by genotype (Table S2).

Tobacco Use Phenotypes: The paper uses three measures of tobacco use available in the survey or laboratory portions of the NHANES examination. Two survey questions were asked of all adults. “Do you smoke cigarettes now?” The proportion stating “yes” was 25%. “About how many cigarettes do you smoke per day?” The mean number of cigarettes for the full sample was 3.73 and among smokers, the mean number of cigarettes was 15.2. The laboratory based measure used was the serum cotinine level (ng/ml). Cotinine is an alkaloid found in tobacco and a metabolite of nicotine with an in vivo half-life of approximately 20 hours and typically detectable for up to a week after tobacco use. The level of serum cotinine is proportionate to the amount of exposure to tobacco smoke (including secondary/passive smoke) (S7). The mean level in the sample was 68.4 (139.3). The typical cut-off for the cotinine level that reflects a smoker is 10 ng/ml. Using this cut-off, the concordance rate between self reported and cotinine assessed smoker status was 92%.

**References**

S1. Mineur YS, Picciotto MR (2008). Genetics of nicotinic acetylcholine receptors: relevance to nicotine addiction. *Biochem Pharmacol* 75: 323–333.

S2. Saccone SF, Hinrichs AL, Saccone NL, Chase GA, Konvicka K, Madden PA et al. Cholinergic nicotinic receptor genes implicated in a nicotine dependence association study targeting 348 candidate genes with 3713 SNPs. Hum Mol Genet 2007; 16: 36–49.

S3. Zeiger, Joanna S., Brett C. Haberstick, Isabel Schlaepfer, Allan C. Collins, Robin P. Corley, Thomas J. Crowley, John K. Hewitt, Christian J. Hopfer, Jeffrey Lessem, Matthew B. McQueen, Soo Hyun Rhee, and Marissa A. Ehringer. The neuronal nicotinic receptor subunit genes (CHRNA6 and CHRNB3) are associated with subjective responses to tobacco. *Hum. Mol. Genet*. (2008) 17(5): 724-734

S4 Greenbaum, L and B Lerer. (2009). Differential contribution of genetic variation in multiple brain nicotinic cholinergic receptors to nicotine dependence: recent progress and emerging open questions. *Molecular Psychiatry*, 14: 912-945

S5. Hoft, Nicole R, Robin P Corley, Matthew B McQueen, Isabel R Schlaepfer, David Huizinga and Marissa A Ehringer. Genetic Association of the CHRNA6 and CHRNB3 Genes with Tobacco Dependence in a Nationally Representative Sample. *Neuropsychopharmacology* (2009) 34, 698–706

S6. Orzechowski and Walker (1999). The Tax Burden on Tobacco. Historical Compilation 1999, vol. 34. Arlington, Va.: Orzechowski and Walker, 1999.

S7. Florescu A, Ferrence R , Einarson T, Selby P, Soldin O, Koren G (2009). "Methods for quantification of exposure to cigarette smoking and environmental tobacco smoke: focus on developmental toxicology". Therapeutic Drug Monitoring 31 (1): 14–30. PMID 19125149.

S8. Dajas-Bailador F. and S. Wonnacott, Nicotinic acetylcholine receptors and the regulation of neuronal signalling, *Trends Pharmacol Sci* **25** (2004), pp. 317–324

1. <http://www.cdc.gov/nchs/nhanes/about_nhanes.htm> [↑](#footnote-ref-1)
2. The full list of genotypes is available online: <http://www.cdc.gov/nchs/data/nhanes/genetics/restricted_snplist_NHIII.pdf> [↑](#footnote-ref-2)
3. See (S8) for general overview of neuronal nicotinic acetylcholine receptors. [↑](#footnote-ref-3)
4. (S1) discuss that nicotine is believed to act in part through activation of the mesocorticolimbic system and that activation of nAChRs on dopaminergic neurons of the ventral tegmental area (VTA) increases their firing rate and stimulates dopamine release from their terminals in the nucleus accumbens. Interestingly lesions of nicotinic antagonists into the VTA have been shown to prevent the development of behaviors related to nicotine addiction, particularly using animal models. [↑](#footnote-ref-4)
5. <http://www.cdc.gov/nchs/nhanes/genetics/collection_dna.htm> [↑](#footnote-ref-5)
